# Factors associated with childhood overweight and obesity in Uganda: a national survey

**DOI:** 10.1186/s12889-021-11567-1

**Published:** 2021-08-03

**Authors:** Quraish Sserwanja, Linet M. Mutisya, Emmanuel Olal, Milton W. Musaba, David Mukunya

**Affiliations:** 1 Programs Department, GOAL, Arkaweet Block 65 House No, 227 Khartoum, Sudan; 2 Maternal and Child Health Project, Swedish Organization for Global Health, Mayuge, Uganda; 3 Yotkom Medical Centre, Kitgum, Uganda; 4 Department of Obstetrics and Gynaecology, Busitema University, Mbale, Uganda; 5 Department of Community and Public Health, Busitema University, Mbale, Uganda; 6 Sanyu Africa Research Institute, Mbale, Uganda

**Keywords:** Prevalence, Obesity, Overweight, Children and Uganda

## Abstract

**Background:**

Childhood obesity is an emerging public health problem globally. Although previously a problem of high-income countries, overweight and obesity is on the rise in low- and middle-income countries. This paper explores the factors associated with childhood obesity and overweight in Uganda using data from the Uganda Demographic and Health Survey (UDHS) of 2016.

**Methods:**

We used Uganda Demographic and Health Survey (UDHS) 2016 data of 4338 children less than 5 years. Multistage stratified sampling was used to select study participants and data were collected using validated questionnaires. Overweight and obesity were combined as the primary outcome. Children whose BMI z score was over two were considered as overweight while those with a BMI z score greater than three were considered as obese. We used multivariable logistic regression to determine factors associated with obesity and overweight among children under 5 years of age in Uganda.

**Results:**

The prevalence of overweight and obesity was 5.0% (217/4338) (95% CI: 4.3–5.6), with overweight at 3.9% (168/4338: 95% CI: 3.2–4.3) and obesity at 1.1% (49/4338: 95% CI: 0.8–1.5). Mother’s nutritional status, sex of the child, and child’s age were associated with childhood obesity and overweight. Boys were more likely to be overweight or obese (aOR = 1.81; 95% CI 1.24 to 2.64) compared to girls. Children who were younger (36 months and below) and those with mothers who were overweight or obese were more likely to have obesity or overweight compared to those aged 49–59 months and those with underweight mothers respectively. Children from the western region were more likely to be overweight or obese compared to those that were from the North.

**Conclusion:**

The present study showed male sex, older age of the children, nutritional status of the mothers and region of residence were associated with obesity and overweight among children under 5 years of age.

## Background

Sub-Sahara Africa is facing an epidemiological transition towards non-communicable diseases (NCDs) such as cardiovascular illnesses and diabetes, with about one third of current disability-adjusted life years (DALYs) attributable to NCDs [1]. The key drivers of the transition are yet to be described, however some documented risk factors include rising socio-economic status, urbanization, physical inactivity, limited and poor dietary choices, cultural misconceptions, and childhood obesity [1–4].

Childhood obesity is an emerging public health burden. Globally over 38 million children under the age of 5 years are affected [5–7]. Children are classified as overweight or obese if their weight-for-height is greater than two or three standard deviations above the age specific median as defined by the World Health Organization (WHO) [7, 8]. Previously, childhood obesity was thought to be a problem of high-income countries, but low- and middle-income countries are now registering higher proportions of overweight and obese children [7, 9]. In the last two decades, Africa has experienced a rise in overweight and obesity among children [7, 10].

Obesity arises from a complex interaction between behavioral, metabolic, environmental, and socioeconomic determinants ranging from changing food systems and reduced physical activity to indiscriminate marketing that promotes obesogenic foods [6, 11–13]. In addition, childhood obesity has been attributed to other risk factors such as high birth weight, maternal obesity, maternal smoking, consanguineous marriage, and poor breastfeeding practices [8, 14–16]. Similarly, the risk of overweight or obesity has been shown to be more prevalent among children attending affluent primary schools as compared to those in rural public schools [17, 18]. This could partly be explained by limited physical activity, excessive sugar consumption and uncensored food adverts that promote obesogenic diets for children in such schools [12, 15, 19].

Tackling childhood obesity is important because it is associated with unfavorable health and socioeconomic consequences. Excessive weight gain is linked to premature mortality and adverse short term and long-term effects on mental health. Furthermore, overweight and obese children have higher risk of early chronic diseases onset, such as diabetes, dyslipidemias, cardiovascular diseases and some cancers [6, 10, 20]. Additionally, they are reported to have lower educational attainment because of poor psychosocial wellbeing [6, 8], higher costs to health systems, and greater financial burden to households [6].

A plethora of studies from Africa have primarily focused on the study of undernutrition and not overweight or obesity among children. The few studies on obesity have mostly come from Nigeria and South Africa, and almost none from other countries in Sub-Sahara Africa [18]. In Uganda, there has been a steady increase in the prevalence of overweight and obesity in the preceding decade [21, 22]. The prevalence of overweight and obesity being highest in descending order, in the Central, Western, Eastern and Northern Regions, respectively [22]. Nevertheless, the distribution and determinants of obesity were not well articulated. Understanding the distribution and determinants of obesity or overweight is vital in designing public health interventions [10, 17]. Therefore, we aimed to determine the factors associated with childhood obesity and overweight in Uganda using data from the Uganda Demographic and Health Survey (UDHS) of 2016.

## Materials and methods

### Study design and participants

UDHS 2016 was a nationally representative cross-sectional study conducted using validated questionnaires. UDHS is a periodical survey that is carried out every 5 years as part of the MEASURE DHS global survey and collects Information on demographic, health and nutrition indicators. The survey was conducted between June 2016 and December 2016 using stratified two-stage cluster sampling design that resulted in the random selection of a representative sample of 20,880 households [22, 23]. The households were randomly selected in two stages: clusters (or enumeration areas) were drawn in the first stage and then a count within each cluster led to a list of households from which was conducted a systematic sampling with equal probability [22]. A detailed explanation of the sampling process is available in the UDHS 2016 report [22]. A systematic random draw was conducted amongst the selected households to choose households whose women/ mothers’ and children’s anthropometric measurements (weight and height) were taken. Anthropometric measurements were done on a subsample of about one-third of households [22]. Weight was taken with an electronic SECA 878 flat scale while a Shorr Board® measuring board was used for height [22]. Children less than 24 months were measured lying down.

Our secondary analysis excluded children whose BMI z-score were missing or was recorded as “Flagged cases”. Flagged cases were defined as more than 5 SD above or below the standard population median (Z-scores) based on the WHO Child Growth Standards [24]. In the children’s dataset, a final weighted sample of 4338 was analyzed after excluding flagged cases and those with missing values. Written permission to access the whole UDHS database was obtained through DHS program website at the address https://dhsprogram.com/.

### Variables

#### Dependent variables

The BMI z-scores based on WHO 2006 reference population were used to assess obesity and overweight [25]. Children whose BMI z score was over two were considered as overweight and those with a BMI z score greater than three were considered as obese [25].

#### Independent variables

Independent variables were categorized into children, parents’ and household characteristics that were chosen basing on previous studies [25–27] and availability in the UDHS data base.

#### Parental characteristics

Maternal nutritional status (underweight defined as body mass index (BMI) less than (<) 18.50 kg/m^2^, normal between 18.50 kg/m^2^ and 24.99 kg/m^2^ and overweight or obesity between 25.0 kg/m^2^ and above 30.0 kg/m^2^) [23, 28], mother’s level of education (no education, primary, secondary and tertiary), father’s level of education (no education, primary, secondary and tertiary), mother’s age (15–24, 25–34, 35–49), mother’s marital status (married and not married), mother’s working status (working and not working). Mother’s marital status was excluded from the multivariable model because data on father’s level of education was missing for children whose mothers were not married (separated, widowed and never been in a formal or informal relationship).

#### Household characteristics

Wealth index is a measure of relative household economic status and was calculated by DHS from information on household asset ownership using Principal Component Analysis (categorized into quintiles: richest, richer, middle poorer and poorest) [22], type of residence (urban and rural), number of household members (less than 5 and 5 and above), sex of household head (female and male) and region (North, East, West and Central).

#### Children’s characteristics

Age of the child in months (0–12, 13–24, 24–36, 37–48, 49–59), sex of the child (male and female) and stunting status (categorized as stunted and not stunted) defined as height-for-age Z-score is below minus two standard deviations (− 2 SD) from the median of the reference population [22].

### Statistical analysis

We used the SPSS analytic software version 25.0 Complex Samples package for this analysis. Weighted data was used to account for the unequal probability sampling in different strata. Frequency distributions were used to describe the background characteristics of the children. Pearson’s chi-squared tests were used to investigate the significant differences between childhood obesity and overweight and the explanatory variables. Bivariable logistic regression was also conducted and we present crude odds ratio (COR), 95% confidence interval (CI) and *p*-values. Variables included in our multivariable model were determined a priori during literature review [29]. All variables in the model were assessed for collinearity, which was considered present if the variables had a variance inflation factor (VIF) greater than 10. However, none of the factors had a VIF above 3. Sensitivity analyses were done excluding underweight children so that a comparison was made between overweight and obese children and normal weight children. We also conducted sensitivity analyses excluding children less than 2 years since some literature suggests that BMI is not an appropriate index for that age group.

## Results

Of the 4338 children, 5.0% (217/4338) (95% CI: 4.3–5.6) were overweight or obese (overweight 3.9% (95% CI: 3.2–4.3) and obesity 1.1% (95% CI: 0.8–1.5). Mean age of the children and that of their mothers was 28.34 months (SD 17.20) and 28.80 years (SD 6.83) respectively. Boys were slightly more than girls. The highest number of children were those less than 1 year. Majority of the children resided in rural areas and belonged to households with five or more members. The mean weight, height, number of household members and BMI z-score were 11.5 kg (SD 3.57), 83.5 cm (SD 14.0), 6 members (SD 2.7) and 0.20 (SD 1.19) respectively. More detailed characteristics of study participants are shown in Table 1.

**Table 1:** Background characteristics of children under 5 years as per the 2016 UDHS

Characteristics	***N*** = 4338	%
**Mother’s BMI**^**a**^		
Underweight	349	8.0
Normal	2948	68.0
Overweight	1034	23.8
**Residence**		
Urban	883	20.4
Rural	3455	79.6
**Region**		
North	871	20.1
West	1128	26.0
East	1225	28.2
Central	1115	25.7
**Household size**		
5 and above	3091	71.2
Less than 5	1247	28.8
**Mother’s working status**		
Not working	918	21.2
Working	3420	78.8
**Mother’s marital status**		
Not Married	589	13.6
Married	3749	86.4
**Mother’s education Level**		
No Education	488	11.2
Primary Education	2673	61.6
Secondary Education	901	20.8
Tertiary	277	6.4
**Wealth Index**		
Poorest	986	22.7
Poorer	891	20.5
Middle	854	19.7
Richer	769	17.7
Richest	839	19.3
**Age Mum years**		
15–24	1372	31.6
25–34	1982	45.7
35–49	984	22.7
**Age of child months**		
Less than 12	1017	23.4
13–24	920	21.2
25–36	859	19.8
37–48	842	19.4
49–59	700	16.1
**Child’s stunting status**		
Yes	1214	28.0
No	3124	72.0
**Head Household**		
Female	1115	25.7
Male	3223	74.3
**Sex of child**		
Female	2148	49.5
Male	2190	50.5
		55
		5
		66
		3f
		444
		888
**Fathers’ education**^**b**^ **N**	148	
No education	266	6.1
Primary	1309	
	1981	45.7
	78	
	66	
	66	
	66	
Secondary	972	22.4
Tertiary	422	9.7
**Child nutritional status**		
Obesity	49	1.1
Overweight	168	3.9
Not overweight	4121	95.0

^a^Missing 7, ^b^missing 697

### Factors associated with childhood obesity or overweight

Region, BMI status of the mother and sex, stunting status, and age of the child were significant factors in the bivariable analysis. Results from multivariable logistic regression (Table 2) showed that mother’s nutritional status, sex of the child, and child’s age were associated with childhood obesity and overweight. The association with region (western) were imprecisely significant. Boys were more likely to be over nourished (aOR = 1.81; 95% CI 1.24 to 2.64) compared to girls. Children who were younger (36 months and below) and those with mothers who were overweight or obese were more likely to have obesity or overweight compared to those aged 49–59 months and those with underweight mothers respectively. The imprecise association with region showed that children from the western region (aOR = 1.65; 95% CI 0.98 to 2.80) were more likely to be overweight or obese compared to those from the Northern region.

**Table 2:** Association between different factors and obesity and overweight among under 5 children in Uganda

Characteristics	Bivariable analysis COR (95%CI)	***P***-value	Adjusted model. AOR (95%CI)
**Mother’s BMI**		0.019	
Underweight	1		1
Normal	**2.59 (1.21–5.55)** ^*^		2.05 (0.94–4.50)
Overweight	**3.01 (1.39–6.50)** ^*^		2.72 (1.22–6.08)^*^
**Sex of child**		**<** 0.001	
Female	1		1
Male	2.07 (1.47–2.90)^*^		1.81 (1.24–2.64)^*^
**Region**		0.004	
North	1		1
West	1.81 (1.21–2.69)^*^		1.65 (0.98–2.80)
East	1.12 (0.71–1.76)		1.09 (0.65–1.84)
Central	0.94 (0.57–1.55)		0.60 (0.29–1.24)
**Residence**		0.218	
Rural	1		1
Urban	0.77 (0.51–1.38)		0.70 (0.41–1.18)
**Stunting status**		0.012	
No	1		1
Yes	1.51 (1.10–2.08)^*^		1.40 (0.94–2.08)
**Working status**		0.152	
Not working	1		1
Working	1.34 (0.90–2.00)		1.22 (0.80–1.87)
**Age of the child months**		< 0.001	
49–59	1		1
37–48	2.07 (1.04–4.15)^*^		1.82 (0.88–3.78)
25–36	3.80 (1.96–7.37)^*^		3.38 (1.68–6.82)^*^
13–24	4.30 (2.30–8.04)^*^		3.70 (1.91–7.17)^*^
Less than 12	3.42 (1.80–6.48)^*^		2.79 (1.44–5.42)^*^
**Father’s Education**		0.292	
No Education	1		1
Primary Education	1.82 (0.91–3.66)		1.43 (0.68–3.01)
Secondary Education	1.44 (0.71–2.94)		1.31 (0.61–2.81)
Tertiary	1.33 (0.55–3.24)		1.08 (0.36–3.23)
**Wealth Index**		0.410	
Poorest	1		1
Poorer	1.48 (0.95–2.32)		1.39 (0.85–2.27)
Middle	1.42 (0.88–2.29)		1.19 (0.67–2.14)
Richer	1.50 (0.90–2.50)		1.25 (0.67–2.31)
Richest	1.18 (0.69–2.03)		0.95 (0.41–2.22)
**Age Mum years**		0.265	
15–24	1		1
25–34	0.87 (0.60–1.26)		0.88 (0.56–1.39)
35–49	0.70 (0.45–1.08)		0.90 (0.53–1.52)
**Household size**		0.308	
5 and above	1		1
Less than 5	1.18 (0.86–1.64)		0.90 (0.60–1.33)
**Household Head**		0.127	
Female	1		1
Male	0.76 (0.53–1.08)		0.74 (0.48–1.15)
**Mother’s marital status**		0.496	
Not married	1		1
Married	0.84 (0.51–1.38)		–
**Mothers’ education level** **N**		0.346	
No education	1		1
Primary	1.51 (0.90–2.53)		1.25 (0.69–2.24)
Secondary	1.54 (0.84–2.84)		1.34 (0.62–2.86)
Tertiary	1.89 (0.85–4.19)		2.39 (0.77–7.43)

*Significant at *p*-value < 0.05

### Sensitivity analysis

After excluding underweight children from the analysis sample, the prevalence of overweight or obesity was 4.6% (138/3001) (95% CI: 3.7–5.2) with overweight at 3.7% (95% CI: 2.9–4.2) and obesity 0.9% (95% CI: 0.6–1.3). Only sex and age of the child were the significant factors. The association between sex of the child with being overweight or obese was stronger compared to the primary analysis with boys (AOR: 2.10 (95% CI: 1.32–3.34) being twice more likely to be overweight or obese compared to the girls. Younger children less than 12 months (AOR: 2.31 (95% CI: 1.03–5.18), 13–24 months (AOR: 4.15 (95% CI: 1.87–9.19) and 25–36 months (AOR: 3.37 (95% CI: 1.35–8.40) were more likely to be overweight or obese compared to the older children aged 49–59 months. After excluding children below 24 months, the prevalence of overweight or obesity was 4.0% (99/2487) (95% CI: 3.1–4.7) with overweight at 3.5% (95% CI: 2.7–4.1) and obesity 0.5% (95% CI: 0.3–0.9). Sex and age of the child were still the only factors significantly associated with overweight or obesity. Children less than 36 months (AOR: 3.44 (95% CI: 1,72–6.90) were more likely to be overweight or obese compared to their older counterparts (49–59 months). Boys (AOR: 2.30 (95% CI: 1.38–3.84) were more likely to be overweight or obese compared to girls.

## Discussion

This study provides evidence on the factors associated with childhood obesity and overweight in Uganda from the UDHS conducted in 2016. We found that maternal overweight and obesity, age and sex of the child as well as region of residence were associated with the child’s overweight or obesity status. For instance, children of mother’s who were overweight were almost three (2.7) times more likely to be overweight or obese compared to those whose mothers were underweight.

The strong association between maternal obesity and childhood overweight/obesity has also been demonstrated elsewhere. Two previous studies showed similar findings with this study where the risk of childhood obesity was increased in cases of maternal overweight/obesity both before and during pregnancy, which also predisposed them to obesity in later life [30, 31]. This relationship can be attributed to various plausible mechanisms, mainly genetic and environmental factors, and their interactions. For instance, interaction between genetic factors associated with obesity and obesity-promoting environments enhances the effect of these genes leading to overall increase in body weight. Further, changes in environmental conditions that allow a pregnant woman easier access to energy-dense foods, would also alter the expression of genes related to body fatness [31]. With regard to lifestyle, sedentary behavior coupled with low levels of physical activity (which are exacerbated by poor food choices, favoring high calorie density) further increases their likelihood of obesity [32, 33].

This study demonstrated that boys had a statistically significant higher risk of being overweight or obese compared to girls, which is consistent with the findings of Beatrice J et al. [34] and Dubois et al. [35]. In these studies, the authors found that boys were more likely to be overweight or obese compared to girls. The differences between boys and girls could be explained by their genetic differences (boys generally tend to be bigger than girls), metabolism and environmental & behavioral factors partly explain this association.

Children aged 36 months and below were more likely to be overweight or obese compared to those aged between 49 and 59 months. A study conducted in Cameroon found a similar association [25] where children below 49 months of age had higher odds of being overweight or obese compared to their older counterparts. This association was stronger in younger children (less than 37 months) and was weakened in older children. Toddlers and preschoolers’ total diet and activity level play an important role in determining a child’s weight. As such, older children are more active compared to the younger ones, hence expending more energy.

Regional differences in the odds of being overweight or obese were imprecise in this study. However, some pertinent regional differences have been documented that may play a role in the observed differences. The western region of Uganda, for example, receives more rainfall annually, has higher crop yields hence better food security, and has the second highest GDP per capita [36, 37]. These factors may account for the increased risk of overweight and obesity among children. In contrast, Northern Uganda has some of the poorest and most food insecure sub-regions which could partly be attributed to the fact that the region experienced a long civil war which greatly affected agricultural production [23]. Furthermore, most people in the Northern part of Uganda are pastoralists, having a nomadic lifestyle which may negatively affect agricultural production of food crops and subsequent consumption, which substantially narrows their diet choices [23]. The decreased agricultural production have resulted in decreased food availability and access in this region.

### Strengths

We used a nationally representative sample with data weighted to account for the unequal probability sampling in different strata, so our results are generalizable to all Ugandan children below the age of 5 years. Secondly, our study was adequately powered to study all these associations because of the large sample size from the UDHS.

### Limitations

The lack of other measures of overweight and obesity, such as skin fold thickness measurements, measure of physical activity, analysis of nutritional characteristics such as dietary habits was major limitation. This data/information would have allowed us to assess the development of overweight and obesity. The cross-sectional design is limited by lack of temporality hence causality inferences could not be made. BMI adjusted for-age for children under 2-years is a contested measure because in this age group, BMI is based on Length other than height. BMI for age (calculated with length) in infancy has un unanswered questions however recent studies have found a high level of agreement between BMI for age and length for age and found BMI for age to be more appropriate [38].

## Conclusion

This study established maternal nutritional status (BMI), region of residence, sex and age a child as significant determinants of childhood overweight/ obesity. Failure to observe any significant differences between wealth quintiles implies that strategies to improve child nutrition should be applied across all socioeconomic groups. Preventive interventions will also need to consider regional differences in outcomes and context. Efforts should be made to promote healthy weight in mothers across the spectrum of motherhood (prenatal, antenatal, and postnatal), as a healthy mother will likely have a healthy child.

Further studies including nutritional characteristics are needed to understand the association with child age and sex and will help in refining preventive strategies against childhood overweight and obesity in Uganda.

## Data Availability

Access to the DHS data sets is openly available upon requests made to MEASURE DHS on their website (https://www.dhsprogram.com/data/available-datasets.cfm).

## Abbreviations

EA: Enumeration area
AOR: Adjusted Odds Ratio
CI: Confidence Interval
COR: Crude Odds Ratio
DHS: Demographic Health Survey
UDHS: Uganda Demographic Health Survey
OR: Odds Ratio
SD: Standard Deviation
WHO: World Health Organization
BMI: Body Mass Index
GDP: Gross Domestic Product
SPSS: Statistical Package for Social Science
USAID: United States Agency for International Development
Kg: Kilogram
Cm: Centimeter

## References

[1] Gouda HN, Charlson F, Sorsdahl K, Ahmadzada S, Ferrari AJ, Erskine H, Leung J, Santamauro D, Lund C, Aminde LN, Mayosi BM, Kengne AP, Harris M, Achoki T, Wiysonge CS, Stein DJ, Whiteford H (2019). Burden of non-communicable diseases in sub-Saharan Africa, 1990-2017: results from the global burden of disease study 2017. Lancet Glob Health.

[2] Ngaruiya C, Hayward A, Post L, Mowafi H (2017). Obesity as a form of malnutrition: over-nutrition on the Uganda “malnutrition” agenda. Pan Afr Med J.

[3] Barr AL, Young EH, Smeeth L, Newton R, Seeley J, Ripullone K, Hird TR, Thornton JRM, Nyirenda MJ, Kapiga S, Adebamowo CA, Amoah AG, Wareham N, Rotimi CN, Levitt NS, Ramaiya K, Hennig BJ, Mbanya JC, Tollman S, Motala AA, Kaleebu P, Sandhu MS (2016). The need for an integrated approach for chronic disease research and care in Africa. Glob Health Epidemiol Genom.

[4] Nnko S, Bukenya D, Kavishe BB, Biraro S, Peck R, Kapiga S, Grosskurth H, Seeley J. Chronic diseases in North-West Tanzania and Southern Uganda public perceptions of terminologies, aetiologies, symptoms and preferred management. PLoS One. 2015;10(11)e0142194. 10.1371/journal.pone.0142194 PMC4640879 26555896

[5] Chooi YC, Ding C, Magkos F (2019). The epidemiology of obesity. Metab Clin Exp.

[6] Di Cesare M, Sorić M, Bovet P, Miranda JJ, Bhutta Z, Stevens GA, Laxmaiah A, Kengne AP, Bentham J (2019). The epidemiological burden of obesity in childhood: a worldwide epidemic requiring urgent action. BMC Med.

[7] World Health Organisation (2020). Noncommunicable diseases: Childhood overweight and obesity.

[8] Uğraş Dilmen A, Konşuk Ünlü H, Özcebe LH (2019). Evaluation of being overweight/obese and related sociodemographic factors in 0-5 year age group in Turkey: Turkey demographic health survey 2013 advanced analysis. Turk J Med Sci.

[9] Abarca-Gómez L, Abdeen ZA, Hamid ZA, Abu-Rmeileh NM, Acosta-Cazares B, Acuin C, Adams RJ, Aekplakorn W, Afsana K, Aguilar-Salinas CA, Agyemang C (2017). Worldwide trends in body-mass index, underweight, overweight, and obesity from 1975 to 2016: a pooled analysis of 2416 population-based measurement studies in 128·9 million children, adolescents, and adults. Lancet.

[10] Adom T, Puoane T, De Villiers A, Kengne AP (2017). Prevalence of obesity and overweight in African learners: a protocol for systematic review and meta-analysis. BMJ Open.

[11] Weihrauch-Blüher S, Wiegand S (2018). Risk factors and implications of childhood obesity. Curr Obes Rep.

[12] Sahoo K, Sahoo B, Choudhury AK, Sofi NY, Kumar R, Bhadoria AS (2015). Childhood obesity: causes and consequences. J Family Med Prim Care.

[13] Arinda IK, Sserwanja Q, Nansubuga S, Mukunya D, Akampereza P (2021). Factors associated with over-nutrition among men 15-54 years in Uganda: a National Survey. Nutr Metabol Insights.

[14] Marshall NE, Lau B, Purnell JQ, Thornburg KL (2019). Impact of maternal obesity and breastfeeding intention on lactation intensity and duration. Matern Child Nutr.

[15] Navti LK, Ferrari U, Tange E, Bechtold-Dalla Pozza S, Parhofer KG (2014). Contribution of socioeconomic status, stature and birth weight to obesity in sub-Saharan Africa: cross-sectional data from primary school-age children in Cameroon. BMC Public Health.

[16] World Health Organization. Taking action on childhood obesity report. World Health Organization; 2018. p. 1–8. Available from: https://www.who.int/end-childhood-obesity/publications/taking-action-childhood-obesity-report/en/%0A.

[17] Tadesse Y, Derso T, Alene KA, Wassie MM (2017). Prevalence and factors associated with overweight and obesity among private kindergarten school children in Bahirdar town, Northwest Ethiopia: cross-sectional study. BMC Res Notes.

[18] Fruhstorfer BH, Mousoulis C, Uthman OA, Robertson W (2016). Socio-economic status and overweight or obesity among school-age children in sub-Saharan Africa - a systematic review. Clin Obes.

[19] Meko LNM, Slabber-Stretch M, Walsh CM, Kruger SH, Nel M (2015). School environment, socioeconomic status and weight of children in Bloemfontein, South Africa. Afr J Prim Health Care Fam Med.

[20] Chedjou-Nono E, Sap S, Choukem S-P, Ngosso Tetanye I, Nebongo D, Koki Ndombo O (2017). Cardiometabolic profile of obese children in a sub-Saharan African setting: a cross-sectional study. BMC Pediatr.

[21] Kirunda BE, Fadnes LT, Wamani H, Van den Broeck J, Tylleskär T (2015). Population-based survey of overweight and obesity and the associated factors in peri-urban and rural eastern Uganda. BMC Public Health.

[22] Uganda Bureau of Statistics - UBOS, ICF (2018). Uganda Demographic and Health Survey 2016.

[23] Sserwanja Q, Mukunya D, Habumugisha T, Mutisya LM, Tuke R, Olal E (2020). Factors associated with undernutrition among 20 to 49 year old women in Uganda: a secondary analysis of the Uganda demographic health survey 2016. BMC Public Health.

[24] Zambia Statistics Agency - ZSA, Ministry of Health - MOH, University Teaching Hospital Virology Laboratory - UTH-VL, ICF (2020). Zambia Demographic and Health Survey 2018.

[25] Tchoubi S, Sobngwi-Tambekou J, Noubiap JJ, Asangbeh SL, Nkoum BA, Sobngwi E (2015). Prevalence and risk factors of overweight and obesity among children aged 6-59 months in Cameroon: a multistage, stratified cluster sampling Nationwide survey. PLoS One.

[26] Muthuri SK, Francis CE, Wachira LJ, Leblanc AG, Sampson M, Onywera VO, Tremblay MS (2014). Evidence of an overweight/obesity transition among school-aged children and youth in sub-Saharan Africa: a systematic review. PLoS One.

[27] Danquah FI, Ansu-Mensah M, Bawontuo V, Yeboah M, Udoh RH, Tahiru M, Kuupiel D (2020). Risk factors and morbidities associated with childhood obesity in sub-Saharan Africa: a systematic scoping review. BMC Nutr.

[28] Sserwanja Q, Kawuki J (2020). Prevalence of underweight and associated factors among lactating women in Ethiopia: a mini-review. J Adv Med Med Res.

[29] Lederer DJ, Bell SC (2019). Control of confounding and reporting of results in causal inference studies. Guidance for Authors from Editors of Respiratory, Sleep, and Critical Care Journals. Ann Am Thorac Soc.

[30] Black RE, Victora CG, Walker SP, Bhutta ZA, Christian P, de Onis M (2013). Maternalandchild undernutritionandoverweightinlow-incomeandmiddle-incomecountries. Lancet.

[31] Modi N, Murgasova D, Ruager-Martin R, Thomas EL, Hyde MJ, Gale C, Santhakumaran S, Doré CJ, Alavi A, Bell JD (2011). The influence of maternal body mass index on infant adiposity and hepatic lipid content. Pediatr Res.

[32] Dinsa GD, Goryakin Y, Fumagalli E, Suhrcke M (2012). Obesity and socioeconomic status in developing countries: a systematic review. Obes Rev.

[33] Saunders T (2011). Potential contributors to the Canadian pediatric obesity epidemic. ISRN Pediatr.

[34] Jouret B, Ahluwalia N, Cristini C, Dupuy M, Nègre-Pages L, Grandjean H, Tauber M (2007). Factors associated with overweight in preschool-age children in southwestern France. Am J Clin Nutr.

[35] Dubois L, Girard M (2006). Early determinants of overweight at 4.5 years in a population-based longitudinal study. Int J Obes.

[36] Moyer JD, Rafa M, Sutton P, Wang X (2017). Estimating district GDP in Uganda. Invited Research Paper for USAID.

[37] Turi KN, Christoph MJ, Grigsby-Toussaint DS (2013). Spatial distribution of underweight, overweight and obesity among women and children: results from the 2011 Uganda demographic and health survey. Int J Environ Res Public Health.

[38] Furlong KR, Anderson LN, Kang H, Lebovic G, Parkin PC, Maguire JL, et al. BMI-for-age and weight-for-length in children 0 to 2 years. Pediatrics. 2016;138(1). 10.1542/peds.2015-3809. 10.1542/peds.2015-3809 27343232

[39] Nantale G, Tumwesigye NM, Kiwanuka N, Kajjura R (2017). Prevalence and factors associated with food insecurity among women aged 18-49 years in Kampala slums Uganda; a mixed methods study. J Food Secur.

[40] Okot-Okumu J, Oosterveer P, van Vliet B, Spaargaren G, Oosterveer P (2010). Providing Sanitation for the Urban Poor in Uganda. Social Perspectives on the Sanitation Challenge.

[41] Namy S, Carlson C, Norcini Pala A, Faris D, Knight L, Allen E, Devries K, Naker D (2017). Gender, violence and resilience among Ugandan adolescents. Child Abuse Negl.

